# Advantages of a Point-of-care Digital Rectoscope for Colorectal Surgical Practice: A Video-supported Case Series

**DOI:** 10.1097/SLE.0000000000001372

**Published:** 2025-05-05

**Authors:** David J. Nijssen, Roel Hompes, Wytze Laméris

**Affiliations:** * Department of Surgery, Amsterdam UMC, University of Amsterdam; † Cancer Treatment and Quality of Life, Cancer Center Amsterdam, Amsterdam, The Netherlands

**Keywords:** colorectal, diagnostics, endoscopy, point-of-care, rectoscopy

## Abstract

**Purpose:**

Point-of-care (POC) diagnostic tools can support timely and efficient clinical decision-making. The introduction of a POC digital rectoscope has the potential to enhance colorectal surgical practice by enabling immediate bedside endoscopic evaluation in different settings.

**Methods:**

This case series describes 5 cases, with video documentation illustrating the potential benefits of using a portable digital rectoscope in outpatient follow-up, inpatient postoperative care, and emergency settings.

**Results:**

In a tertiary referral center, POC rectoscopy effectively supported the detection of anastomotic leakage and rectal perforation, response evaluation after neoadjuvant treatment for rectal cancer, and facilitated follow-up after treatment for anastomotic leakage.

**Conclusions:**

POC digital rectoscopy shows promise in enhancing the diagnostic efficiency of colorectal care. Further studies are warranted to evaluate its clinical impact and cost-effectiveness for the illustrated indications.

Point-of-care (POC) diagnostic tools have shown its value in numerous clinical settings. In emergency medicine, the use of these diagnostic modalities, such as POC ultrasound (POCUS), has been widely adopted due to their ability to reduce the time to diagnosis and provide reliable diagnostic accuracy.^1–3^ In primary care settings, POC tests involving blood, urine, or stool sampling are widely implemented and have proven beneficial in terms of both care efficiency and cost-effectiveness.^4,5^ In colorectal surgical practice, diagnosis and management decisions are often based on endoscopic findings. However, patients may experience delays in receiving diagnostic endoscopy due to capacity limits, and endoscopy departments risk becoming overburdened if a higher turnover is required.^6,7^ POC endoscopy enables immediate bedside diagnostic evaluation of the rectum, thereby influencing and accelerating clinical decision-making. The availability of a POC digital rectoscope allows low-threshold endoluminal evaluation in daily colorectal practice. Here, we aimed to illustrate various indications and certain advantages of using a POC digital rectoscope by presenting several clinical cases.

## MATERIALS AND METHODS

**FIGURE 1:**
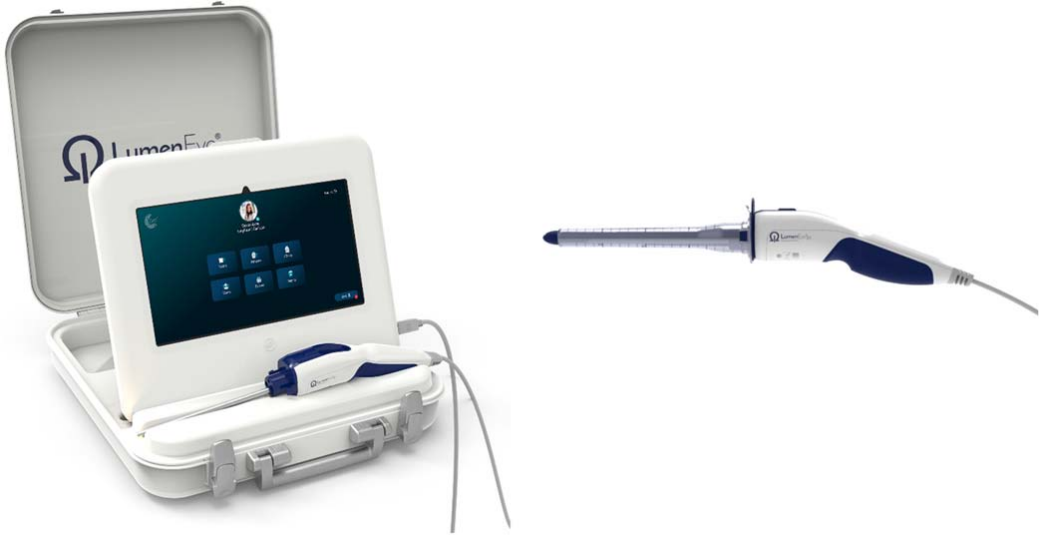
The LumenEye X1 digital rectoscope enabled endoluminal examination of the patients in the case series at point-of-care. Left, The LumenEye connected to the docking station with anonymized video storage. Right, LumenEye with obturator attached ready for clinical use.

Endoscopic examinations were performed using the LumenEye X1 digital rectoscope (SurgEase Innovations), as shown in Figure 1. LumenEye is a portable rigid endoscope that connects to a high-resolution video display, enabling real-time endoluminal vision and bedside image recording. The device features tele-endoscopy capabilities, allowing a second observer or expert to co-assess the endoscopic examination remotely using a wireless network connection. Rectoscopy was routinely performed in the left lateral position. The rigid scope is advanced through a 14 mm disposable rectal tube, which is placed before the examination. If necessary, irrigation of the rectum can be performed through the disposable tube using a 60 mL syringe filled with saline or water. Insufflation can be performed using one hand with an air pump integrated into the handle. Once adequate vision is achieved, the endoscopy images can be recorded and annotated for later reassessment. Video recordings corresponding to the highlighted case reports are included in this report as Supplemental Digital Content, http://links.lww.com/SLE/A487.

### Outpatient Care

#### Case 1: Asymptomatic Fistula 1 Year After Total Mesorectal Excision

**FIGURE 2:**
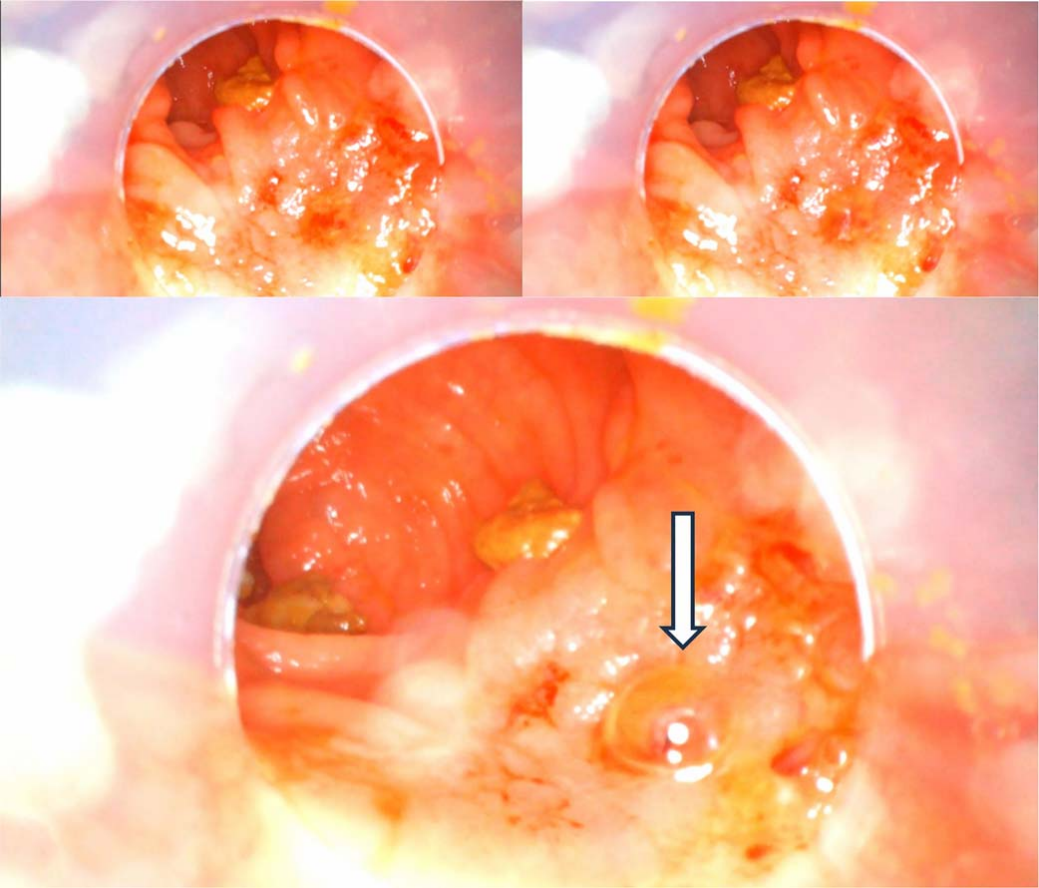
Screenshots taken from the video recording upon discovery of the fistula. The fistula is located on the anterolateral wall of the rectum at the height of the old anastomotic site. The manual insufflator at the rectoscope handles functions similarly to an air leak test, in which the fistula or defect is indicated by air bubbles during insufflation. The full examination video is added in the Supplementary Video file, Supplemental Digital Content 1, http://links.lww.com/SLE/A487.

**FIGURE 3:**
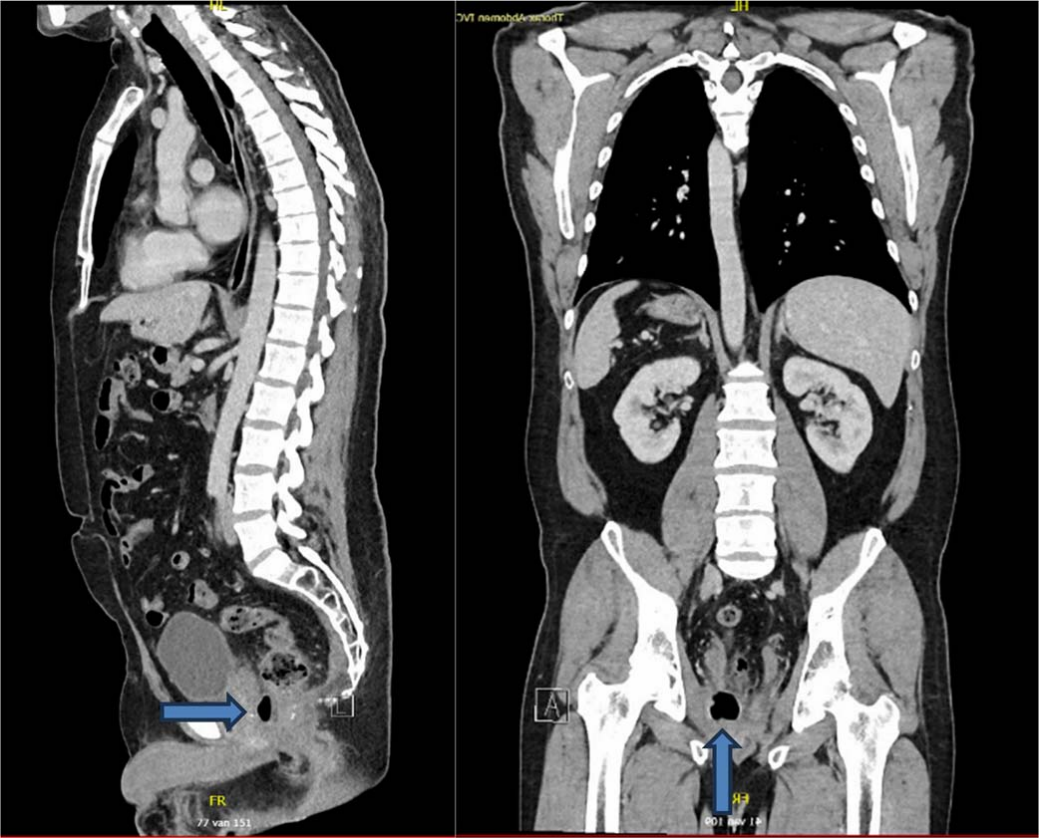
The CT scan of 1 week before the rectoscopy examination in case 1. The blue arrows indicate a cavity containing air and fluid measuring 3 cm in diameter, which connects to the fistula opening found during rectoscopy.

A 42-year-old male patient presented to the outpatient clinic for oncological follow-up 1 year after a transanal total mesorectal excision (TaTME) with neoadjuvant chemoradiotherapy for a ypT3cN2aM0 MRF− EMVI− rectal carcinoma. A diverting stoma was created during index surgery with closure 2 months later. One year after surgery, the patient experienced new onset rectal blood loss. On digital rectal examination, the anastomosis was palpable at 3 cm from the anal verge, with no suspicious findings otherwise. POC rectoscopy revealed an anterolateral anastomotic defect with air leaking upon insufflation of the afferent colon, shown in Figure 2. This finding correlated with the appearance of a chronic sinus adjacent to the anastomosis on a CT scan 1 week prior, as shown in Figure 3. Debridement and primary surgical closure of the sinus cavity were successfully performed 1 month later. A follow-up CT scan after 1 month showed no signs of a persisting sinus.

#### Case 2: Assessment of the Anastomotic Healing After Endoscopic Vacuum-assisted Surgical Closure of the Anastomotic Defect

**FIGURE 4:**
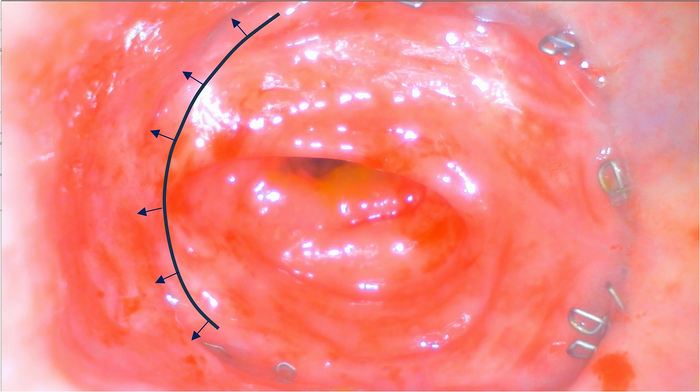
Inspection of the anastomosis 2 and a half months after surgical closure of the defect following endoscopic vacuum therapy. The inspection provided good visualization of the whole anastomosis and indicated proper healing with mucosal alignment at the site of the previous anastomotic detachment indicated by the blue arrows. The full examination video is added in the Supplementary Video file, Supplemental Digital Content 1, http://links.lww.com/SLE/A487.

A 50-year-old female patient visited the outpatient clinic for follow-up after total mesorectal excision (TME) and treatment for subsequent anastomotic leakage. Three months earlier, she had undergone a robot-assisted TME for ycT3N2M0 MRF+ EMVI− rectal carcinoma. Postoperative recovery was complicated by anastomotic leakage, which was initially suspected due to rising CRP levels and confirmed by a CT scan with rectal contrast. In response, the patient received a diverting ileostomy with a colon washout, followed by endoscopic vacuum therapy to manage the leak. After 5 vacuum sponge changes, the defect was surgically closed transanally 18 days after the leak was first detected. A flexible sigmoidoscopy 2 weeks later showed no signs of a persistent leak. However, during oncological follow-up, a contrast CT scan revealed a small air bubble on the anterior side of the anastomosis. To investigate further, POC rectoscopy was performed, which ruled out any persistent clinical leak and confirmed proper healing of the anastomosis (Fig. 4). The patient was scheduled to undergo stoma reversal.

#### Case 3: Response Evaluation After Neoadjuvant Treatment for Rectal Cancer

**FIGURE 5:**
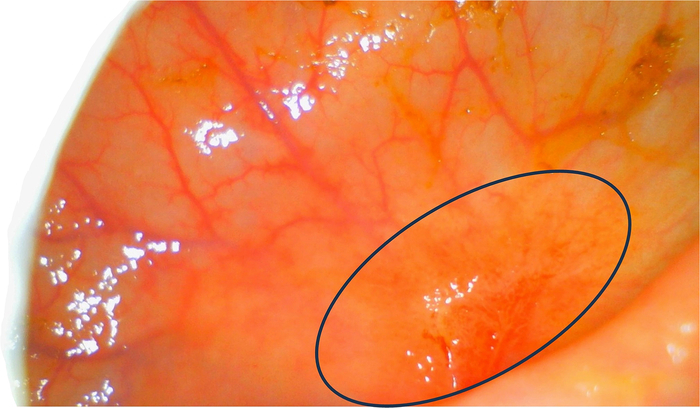
Close-up image of the old tumor site marked by the black oval. No residual signs of malignancy after neoadjuvant therapy, indicating a complete clinical response. The full examination video is added in the Supplementary Video file, Supplemental Digital Content 1, http://links.lww.com/SLE/A487.

A 54-year-old female patient presented to the outpatient clinic for follow-up as part of a wait-and-see policy after receiving short-course neoadjuvant radiotherapy (5 × 5 Gy) for ycT3bN1bM0 MRF− EMVI− rectal carcinoma. The response to neoadjuvant treatment was assessed during this visit, 9 weeks after the last radiotherapy session. During the digital rectal examination, no residual tumor was felt, which had previously been located 9 cm from the anal verge on the anterior side of the rectum. The tumor response was further assessed using POC rectoscopy. At the anterior wall of the rectum, the old tumor site showed minor mucosal whitening surrounded by telangiectasia, without disruption of the mucosal integrity (shown in Fig. 5). The image was consistent with a complete clinical response (cCR).^8^ These findings were in accordance with an MRI performed before the outpatient visit. The patient was monitored further in a wait-and-see protocol.

### Inpatient Care

#### Case 4: Anastomotic Inspection in the Early Postoperative Course After Rectal Cancer Resection

**FIGURE 6:**
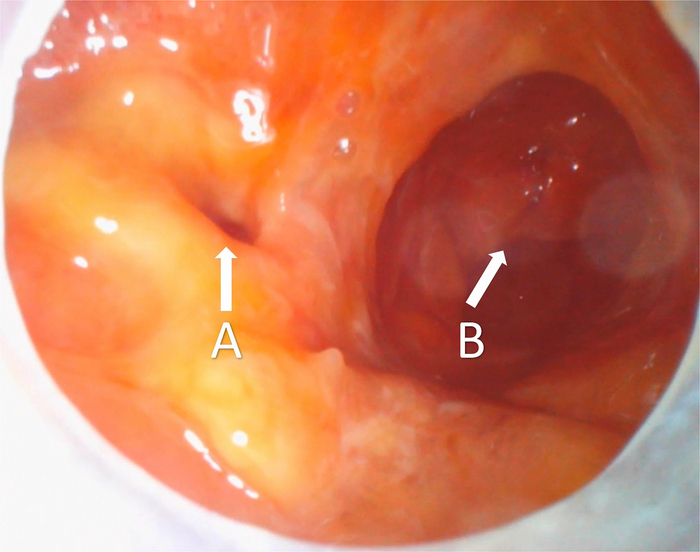
Rectoscopy images from the 65-year-old male 4 days after a total mesorectal excision with the creation of a diverting ileostomy. The examination revealed a clear anastomotic defect, which was not clearly indicated by clinical signs. The defect was treated by endoscopic vacuum therapy. A, Anastomotic defect. B, Afferent bowel. The full examination video is added in the Supplementary Video file, Supplemental Digital Content 1, http://links.lww.com/SLE/A487.

A 65-year-old male underwent a TaTME for a cT3abN0M0 MRF− EMVI− rectal carcinoma without neoadjuvant treatment. Given the intraoperative difficulties requiring conversion to open surgery and vascular comorbidity (diabetes type 2 with end-organ damage), a diverting ileostomy was created. As per protocol the serum C-reactive protein (CRP) levels were determined on postoperative days 3, 4, 170, and 120. There were no clinical signs of pelvic sepsis. Before discharge on postoperative day 5, POC bedside rectoscopy was performed at the ward to assess the integrity and healing of the anastomosis 5 days after surgery. POC rectoscopy revealed an anastomotic defect measuring 1 cm anteriorly, as shown in Figure 6. In the absence of clear clinical signs, the patient would normally have been discharged if no POC system had been available. The anastomotic leak was actively treated by endoscopic vacuum-assisted surgical closure (EVASC).^9^ Closure of the defect was attempted after 16 days; however, the leak persisted. After the second course of endoscopic vacuum therapy, surgical closure of the anastomotic defect was successfully achieved on postoperative day 36.

### Emergency Care

#### Case 5: Perforation of the Rectum

**FIGURE 7:**
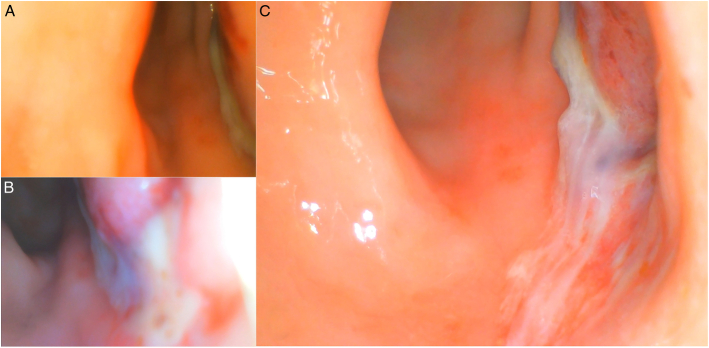
Image A depicts the initial sight of pus and mucus after the introduction of the endoscope at the site of the perforation. Image B provides a close-up view of the defect, showing a clear leakage of purulent fluid around a perforation approximately 7 centimeters from the anorectal junction. Image C illustrates the location of the defect after lavage, with residual drainage of purulent fluid at the site of the perforation. The examination video is shown in the Supplementary Video file, Supplemental Digital Content 1, http://links.lww.com/SLE/A487.

A 73-year-old female presented to the emergency department with alternating diarrhea and constipation, anal pain during defecation, and fever. The patient was treated with chemotherapy for diffuse large B-cell lymphoma. A week prior, she was discharged from hospitalization after treatment with antibiotics for neutropenic fever of unknown origin with positive blood cultures for a multidrug-resistant *E. coli* bacteremia. During that admission, a CT scan had indicated a minor perirectal fluid collection. A previous rectal enema possibly caused the perforation, as the patient reported pain after the enema at history taking. Laboratory results indicated elevated C-reactive protein levels (108) and leukocyte count (12.9). POC rectoscopy was performed by the attending surgeon in the emergency department. A small dorsolateral defect with discharge of pus and air bubbles was detected 8 cm from the anus, shown in Figure 7. The patient was hospitalized and underwent emergency surgery with the creation of an end colostomy.

## DISCUSSION

This case series demonstrates the clinical benefits of POC digital rectoscopy in different colorectal practice settings and highlights the efficiency that can be gained by using a POC endoscopic system. It allows for the immediate evaluation of patients with rectal blood loss, endoscopic response to neoadjuvant therapy, anastomotic integrity after primary rectal surgery and surgical anastomotic repair, and detection of rectal perforations. The continuous availability of endoscopy at the bedside facilitates prompt diagnostic evaluation of disease entities that are easily missed or prone to diagnostic delays. Furthermore, the ability to perform luminal assessments at all times in different clinical scenarios (emergency unit, outpatient clinic, surgical ward, or even ICU) enhances clinical decision-making in surgical practice.

Several digital video rectoscopy systems are currently available on the market, but they have rarely been described in previous reports.^10–12^ Most systems must compromise either mobility or the ability to adequately visualize the entire rectum. This makes their use beyond primary care, where flexible endoscopy is not directly available, less impactful. One advantage of the POC system used in this series is its complete integration into a small mobile unit, which makes it easily transportable throughout the hospital. The examination can be performed at the bedside without assistance or patient transport. Moreover, in the first feasibility study, the diagnostic findings obtained using the POC system were highly correlated with the findings of flexible endoscopy.^13^ Another potential advantage is the hospital cost of endoscopic examination. Compared with formal flexible endoscopy, using a POC system potentially saves the purchase costs, personnel, and overhead costs of the endoscopy unit. Conversely, many POC systems utilize disposables discarded after each examination, causing a continuous need to buy new disposables and related environmental burdens. A comparison of cost-effectiveness has not yet been conducted; however, it is necessary for a fair assessment.

POC endoscopy can hold significant value across different levels of care. For instance, in primary care, prompt evaluation of patients with rectal bleeding can accurately identify rectal pathology and reduce referrals.^5^ Indeed, it could also play a role in screening for malignancies; however, the accuracy of detecting malignant lesions requires further investigation, and the capability to perform a biopsy is necessary in many cases. The ability to perform biopsies by introducing a working channel to POC systems would be a highly valuable addition to colorectal practice.

The use of a portable endoscopic device, as employed in this series, also carries certain limitations. First, the scope is a rigid scope, which, compared with flexible endoscopy, has limited maneuvrability and field of view. One of the main advantages of flexible endoscopy is the presence of a working channel that can introduce instruments into the lumen, for example, to perform biopsies during examination. The use of a POC system should, at this time, not replace the use of flexible endoscopy in the endoscopy unit. However, if used in conjunction, this can make the diagnostic process more efficient, potentially avoiding unnecessary referrals and reducing the time to diagnosis. International multicenter studies are currently investigating the diagnostic accuracy and clinical value of POC rectoscopy systems in surgical practice. These studies aim to utilize POC endoscopy to enhance early detection of anastomotic leakage after rectal surgery in the ongoing REAL study,^14^ assess response evaluation after neoadjuvant therapy for rectal cancer, and examine the rectum in patients who are in a wait-and-see follow-up after complete response of rectal cancer (LUMEVAL).^15^ These studies may also provide insights into the potential impact of POC systems on diagnostic efficiency, time to treatment, and cost-effectiveness, helping to identify for which indications their broader implementation could be valuable to patient care.

In conclusion, this case series has illustrated potential advantages and indications for utilizing a POC digital rectoscope in colorectal surgical practice. Prospective clinical studies evaluating the use of such technology in various clinical scenarios are needed to assess its overall value to the diagnostic process, compared with standard care. In addition, the cost-effectiveness should be evaluated in comparison to current gold-standard diagnostics.

## Supplementary Material
